# Medical Extracts

**Published:** 1883-12

**Authors:** 


### Medical Extracts.

---

### Infarctions and Embolism of various Organs.

Professor D. J. Hamilton thus sums up a paper on the above subject:—

"The chief points that I would wish to draw attention to are:—

"1. That the infarctions of the spleen and kidney are due to the blood supply being cut off from the part, and the most common source of this is embolic plugging of the respective arteries.

"2. They are not usually accompanied by hæmorrhage unless in the zone of inflammation which surrounds them, although congestion and punctiform extravasation are possibilities in the early stages.

"3. They are veritable necroses, the necrotic changes within them resembling those which follow the total abstraction of the blood supply in other parts of the body.

"4. They are in course of time absorbed and the formation of a depressed cicatrix follows.

"5. Embolism of the arteries of the brain is accompanied by a similar necrosis, but, as the arteries of the encephalon are terminal only in certain regions, the necrosis of the part supplied by an occluded artery is never so widespread as in the case of the spleen and kidney. Hæmorrhages may occur here owing to the attempt to nourish the part by collateral channels.

"6. The hæmorrhagic infarction of the lung is simply an apoplexy due to various causes, but by far the most common is rupture of capillary vessels unduly distended by regurgitant pressure from valvular disease of the heart. Its wedge shape is caused by the shape of the bronchus and air-vesicles in which the effused blood is contained, and not by the distribution of a terminal branch of the pulmonary artery. The lesion usually has nothing to do with pulmonary artery embolism, but a hæmorrhage from any cause, if situated at the periphery of the lung, will have the usual characters of a hæmorrhagic infarction.

"7. The blood in these pulmonary infarctions is usually absorbed, and no trace of their former existence remains."—*Liverpool Medico-Chirurgical Journal*, July, 1883.

### The Diagnosis of Movable Kidneys.

"It is an evidence of the minuteness and care with which modern medical science attacks the various problems before it when we find a whole book devoted to the diagnosis of 'l'ectopie rénale,' or misplaced movable kidney. Dr. Fréderic Buret is the author of such a work,* and it is a contribution of practical value to physicians. Although less than one hundred cases of movable kidney have been, so far, reported, it is a trouble which is no doubt much more frequent than is supposed. Such is the opinion of Buret, and Dr. William Roberts, and others, and it is strongly supported by the investigations of Oppolzer, who, in a series of five thousand five hundred patients, found that twenty-two had movable kidneys, giving a proportion of one in two hundred and fifty. It is not improbable, as Roberts says, that many cases of obscure abdominal pain and of gastro-enteric disturbance are due to this cause. Movable kidney occurs in women much oftener than in men, the proportion being as six to one. It is generally an acquired trouble, and its existence is due chiefly to parturition, tight-lacing, sympathetic renal congestion during menstruation, and violent exercise or injury. The most prominent symptoms are a dragging pain in the loin, and gastro-intestinal disturbances, nervous symptoms, hysteria, and hypochondriasis may also be provoked by it. Epigastric pulsation is often present, but the only certain evidence of the trouble is, of course, the presence of a movable tumor which can be felt.

"The chief value of Dr. Buret's work is in the collection of cases which he has made illustrating mistakes in diagnosis. These cases, fifty in all, he divides into three classes: 1st, those in which no tumor was recognised, and no idea of the real trouble was obtained; 2nd, those in which a diagnosis was vaguely formulated; 3rd, those in which a tumor was discovered but its nature not recognized.

"In the first class he cites fifteen cases. These had been treated as cases of crural, or lumbo-abdominal neuralgia, of renal colic, hepatic colic, *embarras gastrique*, hysteria, and chronic peritonitis.

* Du Diagnostic de l'Ectopie Rénale, par le Dr. Frédéric Buret, Aux Bureaux du Progrès Medicale, pp. 93. Paris, 1883.

"In the second class the trouble had been mistaken for 'abdominal tumor' and 'affection of the liver.' Moxæ were applied in some cases and operation suggested.

"In the third case a diagnosis was made of biliary obstruction, enlarged liver, tumor of right lobe of the liver, cancer of the liver, enlarged gall-bladder, biliary calculus, cancer of the pylorus, displaced spleen, and ovarian cyst.

"The list thus given sufficiently illustrates both the inventive imagination of the doctor and the various symptoms which renal ectopia may produce."—*The Medical Record* (New York), July 21st, 1883.

### Worms.

Dr. D. P. Duncan says "Some members of our profession still cling with bull-dog tenacity to the opinion that worms do not affect the health of children, and that they are natural to them. The latter may or may not be true, but when they accumulate in the intestines they produce the same disturbance that any foreign, indigestible substance would do. We find the picking of the nose, swollen lower eye-lids, restlessness in sleep, groaning, gritting teeth, starting, and lastly, spasms.

"Worms kill more children than teething; and when you find the above symptoms with a strawberry tongue and a fever, which will attack several times daily, going off as frequently in cold sweats, you can swear that you have a case of worms, and had as well prepare and attack them.

"Now as to the best means of getting rid of them. I use the fluid extract of senna and spigelia in teaspoonful doses for patients of 8 or 10 years of age, and less in proportion, night and morning, for three nights and days, following this up each morning with a good dose of castor oil, provided the S. and P. does not act. Then wait three days, and again institute the same proceedings, and for the same length of time.

"This treatment is for the lumbricoid. For the oxyuris, or 'thread worm,' I use any bitter infusion by enema, sulph. quinine, or the mur. tr. iron suitably weakened, followed by an enema of common salt and milk—warm water half an hour afterwards, which will destroy and expel them.

"The symptoms of the presence of the worm are the same as the former, with the exception that in the latter you will find the sufferer scratching the anus. If every practitioner will use these he will be gratified by the restoration to immediate health of many a little sufferer who would otherwise linger in sickness for many months and perhaps eventually die."—*Therapeutic Gazette* (Detroit), October, 1883.

### Two cases of Rupture of the Heart.

At the meeting of the New York Pathological Society, on October 24th, 1883, Dr. Ferguson presented the specimens, the first one of which was removed from a man who had had an attack of dyspnœa and about two hours afterwards was found dead in the water-closet. The body was well nourished; the kidneys were atrophied, giving well-marked signs of diffuse nephritis; the lungs were congested; the pericardium contained a large amount of blood; the heart-walls were pigmented and showed signs of general myocarditis. Near the apex in the left ventricle was a rupture three-quarters of an inch in length.

The other specimen belonged to a man who had received an injury on the back of the hand, tearing away the soft structures and exposing the extensor tendons. The wound was dressed antiseptically, and the patient did well the first three weeks, but the muscles of the face and neck and thorax then became contracted, breathing was interfered with, and the patient died in one of the tetanic convulsions. At the autopsy the brain and cord were found congested, the lungs œdematous and congested; the pericardium contained a large amount of blood; the heart was normal, except that in the right ventricle there was a small rupture, admitting a probe. In the one case, therefore, the rupture was due to disease of the cardiac muscle complicated by diffuse nephritis; in the other, to the convulsions of tetanus.—*Philadelphia Medical Times*, November 3rd, 1883.

### Disease Germs: their Vitality; Means of rendering them Innoxious.

M. Paul Bert, President of the Paris Society of Biology, in discussing the question of disease germs recently before that society, said that he had received numerous letters from persons engaged in handling mercury who believed that their occupation afforded them immunity from contracting cholera. Workers in copper are said to enjoy a like immunity from the disease. These supposed facts led M. Bert to seriously question whether there did not exist for each sort of disease germ a destructive agent in the presence of which the germ was unable to develop. M. Davaine, with the view of answering this same question, instituted a series of experiments upon the bacteria of anthrax. M. Bert, with M. Capitan, undertook a similar series of experiments upon the virus of glanders. This virus, "sown" in solutions of sulphate of copper, bichloride of mercury, chloride of gold, or in oxygenated [? ozonized] water, was not fruitful; on the contrary, its development was rapid in solutions of permanganate and of carbonate of potassium.

A first question relative to the vitality of disease germs is, then, one of the medium in which they find lodgment. But the question is also pertinent if, even in a hostile medium, the germ cannot multiply if the quantity of the virus is considerable, or if the medium which receives it is not up to the standard strength, as for example, an organism that has been overworked and exhausted. M. Bouley observed that it was a matter of daily observation that jaded animals were more liable than others to contract glanders or other contagious diseases. The experiments of M. Chauveau on sheep proved that large quantities of virus would avail when small quantities were inert. He produced anthrax in animals by the introduction of virus in considerable quantities when smaller amounts introduced into the same animals had failed to generate the disease.—*New York Medical Journal*, November 3rd, 1883.

### The Symptomatology of Aortic Aneurisms.

Dr. J. Graham Brown, in writing on an article on this subject by Guttmann (*Zeitschrift f. klin. Med.*, vol. vi. p. 131), says:—"It is well known that in cases of aneurism of the thoracic aorta the pathological process which gives rise to the aneurism—the endaortitis chronica deformans—is liable to attack the aortic valves, and thereby occasion insufficiency. In such cases the symptoms of aortic regurgitation add themselves to those of aneurism—if, indeed, the aneurism give rise to any symptoms at all, which is often not the case. In the patient whose case is recorded in this article, however, the reverse was the case, an aortic aneurism giving rise to all the symptoms of aortic regurgitation in spite of complete integrity of the semilunar valves. The signs and symptoms in connection with the circulatory organs which this patient exhibited were the following:—The apex-beat was strong, diffused, and heaving. The cardiac dulness extended from the third left intercostal space above to the sixth rib below, and from the right border of the sternum on the right to a point slightly outside the nipple on the left. On auscultation, there was to be heard over the body of the sternum, the xyphoid cartilage, and in the second left intercostal space, a loud long-drawn diastolic murmur. At the cardiac apex this murmur became almost inaudible. At the same time, a less loud and short systolic murmur was heard over the sternum, and in the second right and left intercostal spaces. Along with this murmur there was faintly audible a systolic sound. The second aortic sound was inaudible, nor was the second pulmonary sound to be heard in its usual site. At the lower end of the sternum a diastolic sound was faintly heard, but whether it was of aortic or pulmonary origin could not be determined. In the carotid and subclavian arteries the first sound was replaced by a murmur synchronous with the heart systole; the second sound was absent. When the stethoscope was lightly applied over the brachial artery, a diastolic sound was audible, which changed into a murmur when pressure was increased. The same phenomena occurred in connection with the femoral artery, and in that vessel there was further audible, when pressure was still more increased, a double murmur systolic and diastolic. All the arteries pulsated strongly, exhibiting beautiful examples of the *pulsus celer*. The diagnosis arrived at from these data was one of aortic insufficiency, but this was not confirmed on post-mortem examination. There was then found hypertrophy of the left ventricle, and a large aneurism involving the ascending aorta and the arch. The aortic valves were completely normal.

In this case, therefore, all the ordinary signs of aneurism (pulsation, dulness, &c.,) failed, while all these of aortic insufficiency were present, along with full integrity of the semi-lunar valves. Guttmann goes on to analyse the physical signs present:—The diastolic murmur, he believes, must have originated in consequence of the irregularity in the rebound-wave in the aorta which normally closes the semi-lunar valves and causes the second sound. The absence of a second sound in the carotid and subclavian arteries is to be explained by the loudness of the diastolic murmur over the heart. The aneurismal sac allowed sufficient back-flow of blood from the arteries to account for the *pulsus celer;* and in the same way is to be explained the abnormal auscultatory phenomena in the vessels.

Thus it is seen that all the so-called cardinal symptoms of aortic insufficiency may exist without any lesion of the valves, in cases of aortic aneurism where hypertrophy of the left ventricle also occurs.

A very similar case came some time ago under the observation of the writer of this abstract, in which all the signs of aortic insufficiency existed, but in which, on post-mortem examination, the valves were healthy, and an aneurism was found which had given no indications of its presence during life. Such observations must make the diagnosis of aortic incompetence very guarded, but it is to be remembered that the conditions under which it can be thus simulated must occur but rarely."—*Edinburgh Clinical and Pathological Journal*, November 10th, 1883.

### The Pathology of Phthisis and its Laryngeal Complications.

At a meeting of the Pathological Society of Philadelphia, on October 25th, 1883, Dr. Seiler read a paper on the pathology of tuberculosis and phthisis and the laryngeal complications of the two diseases. He defined tuberculosis as a self-infectious disease, manifesting itself primarily by the production of minute neoplasms, called miliary tubercles, which rapidly underwent retrograde metamorphosis ending in caseation, being due to the dissemination of infectious material throughout the lymph channels. This infectious cheesy matter was the product of scrofulous inflammation, and it might remain encapsulated for a long time. He then described the histological characteristics of tubercles, and showed how they might produce consolidation of the lung tissue by exciting secondary inflammation. He defined phthisis as a progressive consolidation of the lung tissue, due to a more or less localized inflammation affecting primarily the apices, followed by retrograde metamorphosis. He thought the different forms and stages, as described by many authors, were merely differences in the severity and extent of the ulcerative process. As ætiological factors, he mentioned the various causes of lowered general vitality, predisposing the respiratory organs to chronic inflammation, including heredity, peripheral nerve irritation, hypertrophic nasal catarrh, insufficient aëration of the blood, etc. Improving the general vitality would prevent an outbreak of the disease, and lead to recovery where too much lung tissue had not been destroyed. Such improvement was to be brought about more by proper feeding and exercise in the open air than by the use of drugs. On the other hand, tuberculosis was always fatal, and treatment was of no avail; but a good deal could be done to prevent the formation of the cheesy deposit by improving the health of scrofulous patients early in life, thus preventing the subsequent outbreak of tuberculosis.

The laryngeal complications of both diseases were then considered in detail, and their differences were pointed out. He stated that the laryngeal lesions never appear prior to the lung disease in phthisis; that they were characterized by a peculiar pallor of the mucous membrane; the tumefaction generally affected the posterior portion of the organ, and the ulcerations were shallow and had a tendency to spread on the surface, and tubercles or cheesy deposits were never found in the tissues of the larynx. In tuberculosis, on the other hand, tubercular deposits had been found in the larynx prior to the lung complications; the mucous membrane was of a livid red color; the tumefactions were more commonly observed in the anterior portions of the larynx; and the ulcerations were deeper, with raised edges, and often extensive destruction of tissue.

Dr. Seiler closed with the remark that the indiscriminate use in our literature of the terms phthisis and tuberculosis in referring to lung disease was calculated to mislead the student, and made careful investigation into the pathology and ætiology of these diseases extremely difficult, if not impossible.—*New York Medical Journal*, November 10th, 1883.

### Surgical Expedients in Emergencies.

In a paper read before the Medical Society of the State of Pennsylvania on May 10th, 1883, Dr. R. J. Levis records some of the hastily-devised resources on which the exigencies of active surgical practice had frequently obliged him to rely. Amongst others he gives the following:—

"The absence of a catheter on one pressing occasion led me to contrive a ready means of evacuating the urine. The recourse was to a piece of iron bell wire, bent double on itself, and the blunt doubled end passed readily through the urethral tract to the bladder. The distention of the urethra by the doubled wire allowed the urine to freely pass between the wires.

"*A female catheter* may be extemporized from a short piece of rye straw, the end of which is to be closely wrapped for a short distance with thread; or the end of the straw may have its sharpness removed by dipping into melted sealing wax. The stem of the ordinary clay tobacco pipe is also efficient for the purpose. Such crude substitutes, when oiled, are readily introduced. . . . "I once, on a pressing occasion, bled a patient at the bend of the elbow, with perfect ease and precision, with but a blunt-pointed and dull pocket knife, by resorting to a simple, convenient expedient. Having put on the usual constricting bandage to distend the veins, I first transfixed the most prominent vein with a fine needle. Thus held securely, it was very easy, with even the dull knife, to cut a valvular incision into the vein, and the blood flowed freely.

"In a case of *hemorrhage from the intercostal artery*, from homicidal stabbing, I arrested the flow immediately by making pressure within the pleural cavity, directly on the vessel, by introducing into the wound the handle of a door-key. The key was then turned transversely so as to make direct pressure, and maintained in that position for some hours, until there was no more tendency to hemorrhage. The same mechanical action might be effected by the similar use of the handle of an ordinary gimlet.

"The hæmostatic action of hot water does not seem to be sufficiently known and appreciated among practitioners. It is so effective, and can be so readily applied, that it may well displace from practice all other hæmostatics. Water at a temperature not beyond tolerance of the immersion of the hand in it, which is a temperature of from one hundred and fifteen to one hundred and twenty degrees, is ordinarily all that is necessary; but in some cases not amenable to treatment by the ligature, a temperature above 160° F., the coagulating point of albumen, may be necessary.

"The facility with which rectal injection can be performed with large quantities of fluids, by hydrostatic pressure, renders not essential the use of a syringe, if a piece of india-rubber tubing long enough can be obtained. The lower bowels may also be distended, in cases of intussusception, by injecting water and carbonic acid gas, forced from the ordinary mineral water bottle or syphon, fitted to the rectal tube.

"For antiseptic use many readily procured substances may well replace carbolic acid. None is so cheap and efficient as that most neglected preventer of putrefaction, sulphurous acid, made simply by exposing water to the fumes of burning sulphur in a close chamber. The antiseptic action of a saturated watery solution of turpentine has also the advantage of convenience of procurement and cheapness. For this purpose turpentine should be kept continually in water and exposed to warmth, and frequently agitated. Diluted alcohol has merits as an antiseptic which have not received proper attention."—*The Polyclinic* (Philadelphia), August 15th, 1883.

### Stretching the Optic Nerve.

Dr. M. Landesberg, in a paper read before the Philadelphia County Medical Society, on June 13th, 1883, records the result of his investigations into the therapeutic value of stretching the optic nerve in conditions of atrophic degeneration:—

"Stretching the optic nerve I have performed twenty-one times in thirteen patients—sixteen times after the following method. The lids are separated by the speculum; the incision of the conjunctiva is made on the insertion of the internal muscle, and the latter secured by a silk ligature. The tendon of the internal muscle is dissected, leaving a small stump on the sclera, in order to facilitate the reunion, and Tenon's capsule and the underlying tissue are loosened from the sclera towards the optic nerve. The eyeball is turned outwards to the utmost by means of fixation-forceps, and a strabismus-hook is passed through the opening of the conjunctiva along the eyeball down to the optic nerve, which is caught from above and stretched 'gently' three or four times. The eyeball is then brought into position, the internal rectus is reattached, and the conjunctival wound is closed by one or two sutures. Finally, a compressive bandage is applied for two to three days.

"In five instances I have performed the operation without tenotomy, by making a slit in the lower and outer part of the conjunctiva near the corneal margin, and passing a strabismus-hook between the external and inferior muscle down to the optic nerve. This procedure is very simple, and more harmless than the other one, but it has the great disadvantage of allowing the optic nerve to slip very easily from the strabismus-hook, especially if the patient does not keep quiet.

"The operation itself was in no instance followed by any bad consequences, either local or general, and the symptoms of reaction were such as we usually observe after a strabismus-operation."—*Philadelphia Medical Times*, August 25th, 1883.

Records of the cases are given, and the results were either improvement or *nil.*

### The Prophylaxis of Ophthalmia Neonatorum.

Dr. A. H. F. Barbour says that in a paper (*Archiv. f. Gynäkologie,* Bd. xxi. Hft. 2) on this subject "Credé sums up his experience in the treatment of this affection. During the ten years 1870-1880, the number of children affected with ophthalmia at the Leipzig Maternity was about 12 to 15 per cent. (during two years it was only 6 to 7 per cent.) During the last four years, in which his special treatment has been carried out, it has fallen to ·25 per cent. in one year, and 0 per cent. in the other three. With regard to its etiology, his conclusions are: (1) that it is caused by a specific virus, which is identical with that of gonorrhoea; (2) that the poison, which is found chiefly in patients affected with granular vaginitis, finds its way into the conjunctiva principally during the second stage; (3) that the normal vaginal secretion does not cause ophthalmia; (4) that the existence of granular vaginitis was only proved in a small percentage of his cases, because several were admitted to the Maternity already in labour; (5) that a prolonged second stage (of more than an hour), as well as an early rupture of the membranes (three hours before the birth of the child) and the birth of large children, favour infection. The period of incubation is usually three, rarely five days. Antiseptic vaginal injections and washing of the child's eyes had been tried, but found insufficient to prevent ophthalmia. This led Credé to experiment with a view to destroying the germ, and he has found that a *two per cent. solution of nitrate of silver* effects this. The eyes are first washed, and then a single drop of the solution is let fall on the conjunctiva; no further attention is required. In some cases, specially premature children, some hyperæmia of the conjunctiva follows; in the majority there is no reaction. As 33 per cent. of the children received as blind into the asylums in Germany and Austria owe their condition to this disease, we are not surprised to learn that Credé's method is being enforced by the Government Board of Health."—*Edinburgh Clinical and Pathological Journal*, November 24th, 1883.

### Iodoform in Corneal and Conjunctival Diseases.

Dr. A. G. Heyl quotes from *Arch. f. Ophthal.*, 1883:—"Vossius gives the following results of considerable experience with iodoform. In a case of gonorrhœal ophthalmia it increased the discharge and swelling; one cornea, which previously was involved, was made worse, and the other, which had been intact, likewise became affected. Only one application was made, when a change of treatment was necessary. In blennorrhœa neonatorum it has no special value. In trachoma it sometimes seemed to do good, but oftener not: upon the granulations it did not act well, but sometimes when acute increase of the pannus would occur the iodoform seemed to relieve. Nor did it prove of value in phlyctenular conjunctivitis, pannus scrofulosis, parenchymatous keratitis. On the other hand, remarkably good results were attained in the treatment of corneal ulcer. Ninety corneal ulcers form the material upon which this statement is based. Especially in the ulcus corneæ serpens was the value of the remedy manifest. Thirty-six cases of this kind were treated: in seventeen of these severe dacrycystitis existed; in one old granulation with profuse purulent secretion. In three cases, Saemisch's operation was performed: in two of these, in spite of this and the iodoform treatment, the whole cornea became involved. In thirty-two of the cases surprisingly favorable results were attained by the iodoform alone. The iodoform was used in powder or in the form of salve. It seemed to produce an anodyne effect, relieving the violent ciliary pain and irritable condition of the eye. [An important practical matter seems to find illustration in this experience,—viz., that the treatment of local infection with septic material must vary with the nature of the infecting germ. Thus certain septic forms of conjunctivitis and keratitis are very much helped by the application of heat. The form of corneal ulcer above referred to, where infection very often comes from the lachrymal sac, is certainly not very susceptible to the action of heat, but seems to require a substance which has a different therapeutic action. H.]"—*Philadelphia Medical Times*, November 3rd, 1883.

### A Fœtus remaining Fifty-six Years without Change in a Case of Extra-Uterine Pregnancy.

The following is quoted from the *Gazette Médicale de Nantes*:—"M. Sappey, in a very interesting communication to the

Academy of Sciences (*Courrier Médical*), reported a case that is probably without parallel in medical annals. A woman at 28 years of age became pregnant, but was not delivered. She carried an abdominal enlargement until she died at eighty-four years. At the autopsy a large tumor was found outside of the uterus, connected with the right Fallopian tube. The wall of the cyst was tough, and its surface calcareous and nodular. After removal the cyst was divided with a saw, and, very much to the surprise of the spectators, a fœtus of about seven months' development was found in its interior. During its long captivity it had been perfectly preserved, not having undergone any alteration. 'It was in the ordinary attitude, the limbs flexed upon the trunk, the head inclined on the thorax. The pupillary membranes were completely developed, and fixed the age at from six to seven months. The cutaneous envelope, the superficial organs, as well as the viscera in the large cavities of the body, all the muscles and soft parts, retained their consistence, their suppleness, and their normal colour. The fœtus, in short, presented all the features of an infant which had just gone to sleep.' This case alone upsets the ordinary views with regard to the calcification and desiccation of the fœtus being necessary for the preservation of a fœtus retained in the abdominal cavity. According to Sappey, 'infants which after their death are retained indefinitely in the abdomen of the mother owe their preservation to the physical conditions of their imprisonment, which give them a refuge from atmospheric germs.' "—*Philadelphia Medical Times*, November 3rd, 1883.

### The Treatment of Neglected Abortion.

Dr. V. Idelson thus writes in the *London Medical Record*, Nov. 15, 1883:—

"In the *Mejdunarodnaia Klinika*, May, 1883, pp. 296–309, Dr. A. A. Muratoff, of Moscow, discusses the management of those cases of abortion in which some portions of the ovum are retained within the womb, and undergo there decomposition, threatening septicæmia. Of course, there may be no question of what to do in such cases. The obstetrician must remove the remnants of the ovum as quickly as possible, and disinfect the uterus. But how is this to be done? How is the uterine cavity to be reached, with its dangerous contents, through the os, which is usually found closed? Of all the means proposed and used for dilating the cervix in similar cases, sponge-tents are regarded by the author as the most harmful, for, 1, they act too slowly when every minute is precious; 2, any discharge absorbed by them rapidly undergoes putrefaction; and, 3, they cause irritation of the cervical walls. Laminaria digitata, also, acts very slowly; besides, it easily falls out from the cervix. Tupelo-root dilates the os much more rapidly than sponge or laminaria. Thus, according to Dr. A. Solovioff, while a tupelo-tent reaches its maximal swelling in four hours, a laminaria-tent, of an equal size, does so only in fifteen to twenty hours (see his article in the *Mediz. Obozr.*, May, 1880); but it is very difficult to find tupelo-roots of larger size.

"Basing his arguments on five years' experience, Dr. Muratoff emphatically recommends dilatation of the uterine cervix by means of a metallic dilator. As the best instrument of this kind, he regards Sims's three-valved dilator. Dr. Muratoff's practice is as follows:—After washing out the vagina with 2 or 3 per cent. of carbolic solution, he freely anoints the instrument with 4 per cent. of carbolised vaseline; he introduces its valved end into the cervix, and begins to turn the screw which serves for opening the valves. The first three or five full turns are made without any pause; but from the moment when the patient has felt a sharp pain, he proceeds with dilatation very slowly, making only half a turn at a time in every ten minutes. As a rule the valves are open to their utmost extent in three or four hours. The dilator is left in this condition for a quarter of an hour, and then closed and withdrawn. During the process of dilatation, the author makes repeated intra-uterine injections of a warm carbolic solution, through a double-current catheter. After the withdrawal of the instrument, bimanual expression of the uterine contents is tried. If one attempt be not sufficient, the remnants of the ovum are without any further delay removed by one or two fingers introduced into the womb.

"The writer eulogises the results given by this method of gradual (but still relatively rapid) dilatation in *all* cases of neglected abortion. The presence of any inflammatory processes in the parenchyma of the uterus or in the vicinity of the organ does not contra-indicate the use of metallic dilators; on the contrary, it vitally indicates instrumental dilatation. The latter inflicts no violence on the cervical tissues, and, accordingly, never gives rise to any considerable reaction. In a few cases, indeed, Dr. Muratoff saw a slight temporary rise of temperature and some increase of pain about the sexual apparatus after the operation. But in a great majority of his cases the dilatation, with subsequent emptying of the womb, was immediately followed by a marked improvement both of the local and of the general conditions: the patient's temperature steadily and quickly fell to the normal level, rigors and pain disappeared, the uterine discharge rapidly lost its offensive odour, and the uterus underwent uninterrupted involution."

### The Ether Douche or Lavement for Local Pain.

Dr. C. H. Hughes writes:—"A paragraph in the *Medical Times* of the 10th of February, 1883, referring to the ether spray in the cure of neuralgia, prompts me to call attention to the fact that ether lavements have been employed by me in all painful surface-affections for many years, whether with or without inflammation, but mainly in neuralgic affection. In facial, sciatica, and cervical neuralgias, no remedy except galvanism has given me such signal satisfaction during the past ten years of my practice in neurology. These lavements will cure some cases of recent origin; they will relieve all. I use the ether douche, not the spray, and Dr. McLane Hamilton is in error in his reference to my treatment of the intense pain of cerebellar abscess by ether spray. In the case referred to, which I reported in 1877 (*Journal of Mental and Nervous Diseases*, October), I simply poured the ether on the head so copiously as to benumb all sensibility and restore a state of ease and mental tranquility to a patient absolutely maddened with pain.

The ether douche or lavement in trigeminal neuralgia is quite uncomfortable to many persons, on account of the unpleasant impression of the ether on the nose and eyes; and when applied to the supra-orbital region great care should be taken to keep the ether out of the eyes by laying the head back and covering the eyes with a handkerchief. If the ether should get in the eyes, the patient should be cautioned not to rub them, but simply to sponge the eyes with cold water and wait patiently till the ether evaporates. The same is true in regard to ether getting in the ears.

There is no need of a spray apparatus for ether. It should be used freely in quantities adequate to the effect desired. It should be poured on the part till relief is obtained. I apply it in this way to the motor regions of the head and down the spine in general or unilateral chorea likewise.

No better agent can be employed for cephalalgia and for acute muscular tremor than the ether douche or lavement. Of late years I have heard of the ether spray, but the ether *douche* or lavement has been with me a most common and efficient agent in the local therapy of pain, especially superficial pain, for more than a decade, ranking with electricity, and better than mechanical vibration."—*Philadelphia Medical Times*, September 8th, 1883.

### Adonis Vernalis.

Dr. C. W. Tangeman thus quotes from *Wien. Med. Blätter*:—

"Adonis vernalis, although used empirically in southern

Russia as a remedy in dropsy, had never been thoroughly investigated until quite recently, when Prof. Botkin made a thorough study of it at his clinic. The following is a short resumé of B.'s elaborate article, which was first published in the *Arch. f. klin. Med.* After a series of experiments it was discovered that only in certain kinds of dropsy, or rather dropsy due to certain causes, in cases where the œdema was due to a disturbance in the compensation and activity of the heart, the remedy acted very satisfactorily. The heart-beat increases in force after the use of adonis vernalis, and the size of the heart rapidly diminishes; the heart sounds and murmurs, especially the presystolic and systolic murmurs in stenosis, are more marked and distinct. The heart rhythm is more regular and somewhat slower; therefore the pulse is slower, and in most cases the pulse wave fuller and stronger. The secretion of urine is markedly increased, amounting to a change of from 300—400 ccm. to 2000—3000 ccm., a ten-fold increase of the watery element. All deposits disappear, specific gravity diminishes and the urine has a very pale color. There is an absolute increase of the chlorides and urates, the body weight diminishes and the œdema disappears rapidly; the dimensions of the liver decrease, cyanosis and dyspnœa disappear, and respiration becomes full and regular.

"In the largest number of cases great relief was experienced; at the end of the first day, complaints were less frequent, and in the course of a few days they disappeared entirely. Adonis vernalis has a good effect on cases also where heart disease is of a secondary nature, following chronic Bright's disease, etc. In cases even where the activity of the kidneys was very low and the œdema was well marked, it was very seldom that adonis vernalis did not give relief, provided the heart action was diminished and the blood pressure lower than normal.

"We must append the history of the following case on account of its extreme interest: A common laborer had been taking adonis vernalis for nearly two months and felt very much relieved; the heart diminished in size; the congestion of the lungs, which was well marked, almost disappearing entirely; the œdema of the legs and the ascites disappeared entirely; palpitation of the heart and dyspnœa diminished so much that the patient was discharged from the clinic and returned to work. The remedy was dispensed in the following way:

℞ Infus. adon. vernal. ex. 4.00 ad. coll. 200.00.

Ol. menth. pip., gtt. 2.

M. S. One teaspoonful every two hours."—*Therapeutic Gazette* (Detroit), October, 1883.

